# Quantification of Tongue Motor Dysfunction in Amyotrophic Lateral Sclerosis Using a Smartphone-Based Task and Deep Learning

**DOI:** 10.3390/s26051498

**Published:** 2026-02-27

**Authors:** Pedro S. Rocha, Duarte Folgado, Vasco A. Conceição, Miguel Oliveira Santos, Mamede de Carvalho

**Affiliations:** 1 Institute of Physiology, and Centro de Estudos Egas Moniz, Faculty of Medicine, University of Lisbon, 1649-028 Lisbon, Portugal; pedro.rocha@medicina.ulisboa.pt (P.S.R.); vasco.conceicao@medicina.ulisboa.pt (V.A.C.); miguel.santos@ulssm.min-saude.pt (M.O.S.); 2 Fraunhofer Portugal AICOS, 1649-028 Lisbon, Portugal; duarte.folgado@aicos.fraunhofer.pt; 3 Department of Neurosciences and Mental Health, ULS de Santa Maria, Hospital de Santa Maria, 1649-028 Lisbon, Portugal

**Keywords:** amyotrophic lateral sclerosis, bulbar dysfunction, deep learning, dysarthria, tongue

## Abstract

Background: Bulbar dysfunction is a major complication of amyotrophic lateral sclerosis (ALS). This study aimed to develop and validate a simple, smartphone-based task for the objective assessment of tongue movements and to examine their association with clinical variables. Methods: 37 ALS patients and 20 age- and sex-matched controls performed a tongue lateralization task, recorded with a smartphone. A deep-learning U-Net++-based model was used for segmentation and feature extraction. The frequency and maximum amplitude of tongue movements were quantified. Clinical measures included the ALS Functional Rating Scale-revised (ALSFRS-r) bulbar sub-scores, tongue fasciculations, jaw jerk, and tongue “spasticity”. Between-group differences and associations between tongue metrics and clinical features were assessed. Results: The U-Net++-based model achieved robust segmentation performance. Patients showed lower tongue movement frequency than controls (0.14 vs. 0.40, t = −9.58, *p* < 0.001). Normalized frequency was associated with dysarthria (t = −3.13, *p* = 0.003) but not dysphagia (t = −1.05, *p* = 0.30). Normalized frequency (t = 2.77, *p* = 0.009) and tongue “spasticity” (t = −2.57, *p* = 0.015) were both associated with speech performance in a multiple-regression model (R = 0.51, adjusted R^2^ = 0.43). Conclusions: Our method provides an objective, minimally invasive measure of bulbar function in ALS, which correlates with clinical ratings and may detect subtle impairments not captured by standard assessments. This approach offers a promising tool for remote monitoring and may support more effective disease management.

## 1. Introduction

Amyotrophic Lateral Sclerosis (ALS) is a fast-progressing neurodegenerative disease with significant clinical variability [1]. Bulbar impairment, including dysarthria and dysphagia, is a hallmark feature of the disease, with approximately one-third of patients presenting bulbar symptoms at onset and most developing them as the disease progresses. Bulbar presentation is associated with shorter survival, faster functional decline, and reduced quality of life [2,3]. Dysarthria has been associated with low mood, withdrawal from activities, and social isolation, and dysphagia can lead to weight loss, malnutrition, aspiration pneumonia and increased risk of hospitalization [4,5,6].

The clinical evaluation of bulbar dysfunction continues to depend largely on semi-qualitative scales. The bulbar sub-score of the revised ALS Functional Rating Scale (ALSFRS-r) remains the most widely used tool for routine clinical assessment [7], providing a coarse measure of deficits in speech and swallowing. However, its sensitivity to subtle or early changes in bulbar function is limited and scores may be confounded by symptom management or cognitive changes.

More invasive (electromyography) or sophisticated techniques, such as electrical impedance myography, neuroimaging, kinematic analyses of articulatory movements, and lingual pressure tests, have identified specific abnormalities in patients with ALS [2,8,9,10]. Yet, these approaches often prove challenging to implement in routine clinical settings and may impose substantial burden on patients—particularly those with an advanced disease. This has created a need for objective, accessible, and minimally invasive assessment methods.

Recent technological advances have enabled a growing field of research using various sensors to objectively quantify dysarthria and dysphagia through analysis of speech, voice, and even cough patterns [11,12,13]. Some studies leverage machine and deep-learning techniques to extract personalized features from these analyses, correlating them with disease progression and bulbar impairment severity. These approaches have gained significant attention due to their automation, adaptability, and ability to integrate multimodal data while detecting patterns that are challenging for humans to identify. This represents a significant step toward more personalized and accessible disease management, enabling remote monitoring and improving patient care from home [14,15,16,17]. Furthermore, some of these technologies have the potential to assist in diagnosis and support clinicians in making more informed decisions [18].

To enhance clinicians’ ability to evaluate bulbar signs, we developed a simple clinical task to assess tongue movements. Tongue observation in ALS is part of the diagnosis setup, being critical for detecting signs of lower neuron involvement (LMN), atrophy, and fasciculation. In addition, most clinicians evaluate voluntary movements (protrusion and lateral movements), which, when abnormal in tongue without atrophy, can indicate upper motor neuron (UMN) involvement [19], equivalent to tongue “spasticity” [20,21].

To address these limitations regarding UMN signs, this work aimed to develop an objective method for assessing tongue movements using a deep-learning segmentation model (U-Net++). We hypothesized that our metrics would be abnormal in ALS patients and that they would demonstrate significant correlations with clinical ratings, such as the dysarthria subscore of the ALSFRS-r, while providing enhanced sensitivity to subtle functional changes not captured by routine clinical assessments. Rather than replacing clinical assessment in cases of overt dysfunction, our goal was to develop a simplified approach capable of identifying subclinical changes in tongue motility.

## 2. Methods

### 2.1. Participants

This was a cross-sectional observational study that included consecutive patients observed in our ALS clinic in Lisbon, diagnosed according to the Gold Coast criteria [22]. Differential diagnoses were excluded through comprehensive neurological, neurophysiological, neuroimaging, and laboratory evaluations. Patients with a history of lung disorders, resting dyspnea, laryngeal injury, upper airway infections, or tongue abnormalities like scars, atrophy, or inability to protrude the tongue were excluded. Additionally, exclusion criteria included significant cognitive impairment that would prevent collaboration with the task or declining to participate. The control group comprised healthy individuals matched for age and sex, most of whom were spouses of patients or staff members of the institution. None had a history of neurological or respiratory disorders. All eligible patients approached for the study consented to participate. Furthermore, the video recordings for all recruited patients and controls were of sufficient quality for inclusion in the analysis. The study protocol was approved by the local research ethics committee of the Centro Académico de Medicina de Lisboa (CAML, Ref. 286/25). All participants provided written informed consent prior to enrollment, and the study was conducted in accordance with the Declaration of Helsinki.

### 2.2. Clinical Evaluation

Demographic and clinical data were collected for all ALS participants, including age, sex, disease duration, and site of disease onset. Functional status was assessed using the ALSFRS-r [7]. Bulbar symptoms were quantified using the ALSFRS-r bulbar subscore, which consists of questions 1 through 3 regarding speech, salivation, and swallowing. Patients with a score lower than 11 were considered to have a relevant bulbar dysfunction. The dichotomous definition of bulbar dysfunction was based on the cohort median and was used for descriptive subgroup visualization only. Primary inferential analyses relied on continuous speech scores to avoid information loss associated with dichotomization. The median-based grouping should therefore not be interpreted as a clinically validated threshold. Presence of dysarthria and dysphagia was defined as scores below 4 on the speech and swallowing items, respectively. In all patients, clinical observation included evaluation of tongue atrophy (exclusion criteria), tongue fasciculations (longer than 1-min observation), tongue protrusion, side-to-side rapid movements, and jaw jerk. Tongue fasciculations defined LMN signs, and limited tongue protrusion or slow side-to-side movements in the absence of atrophy (tongue “spasticity”), or brisk jaw jerk defined UMN signs. All assessments were performed by trained neurologists experienced in ALS care (MOS and MdC).

### 2.3. Task and Metrics

**Figure 1:**
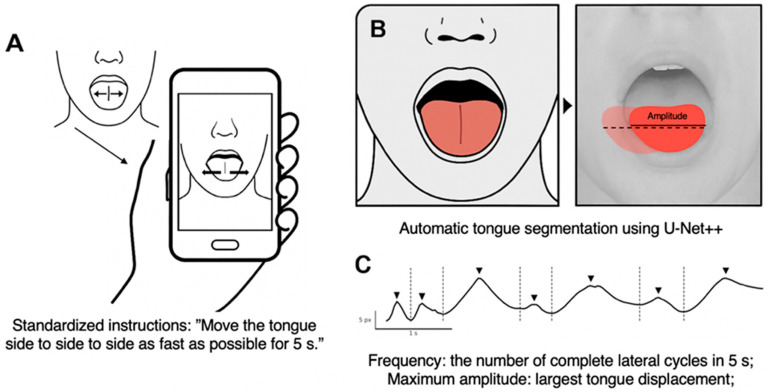
**Overview of the smartphone-based tongue lateralization task.** (**A**) Participants were instructed to move the tongue laterally as rapidly as possible for 5 s while being recorded by a smartphone positioned approximately 15 cm from the mouth. (**B**) A U-Net++ convolutional neural network was used to automatically segment the tongue region in each frame. (**C**) From the segmented trajectories, quantitative features were extracted, including movement frequency (number of complete cycles in 5 s) and maximum amplitude (largest lateral displacement).

The task was designed to provide a clinically feasible assessment of tongue motor function while minimizing patient burden. Participants were seated and instructed to protrude the tongue and to move it from one side to the other, as rapidly as possible for a duration of at least five seconds (Figure 1). They were also asked to keep their head and jaws as still as possible while performing the task. No physical head or chin restraints were used in order to preserve comfort and ecological validity. Instructions were standardized across participants, and verbal encouragement was given during performance if necessary.

Movements were recorded by a trained ALS neurologist using a smartphone (OnePlus BE2013; OnePlus Technology, Shenzhen, China) aligned frontally and positioned approximately 15 cm from each participant’s mouth. Videos were acquired at a resolution of 1080p (progressive scan) and 30 frames per second, under ambient indoor lighting conditions. Default smartphone camera settings, including automatic exposure and white balance, were allowed to ensure ease of deployment and reproducibility in non-specialized settings. The camera field of view was framed to include only the tongue and immediate perioral region, ensuring a consistent region of interest across recordings while preserving participant anonymity. Recordings were visually inspected for quality prior to analysis. Videos were excluded if the tongue was not fully visible throughout the task, if excessive head or jaw movement was present, or if motion blur prevented reliable segmentation.

**Figure 2:**
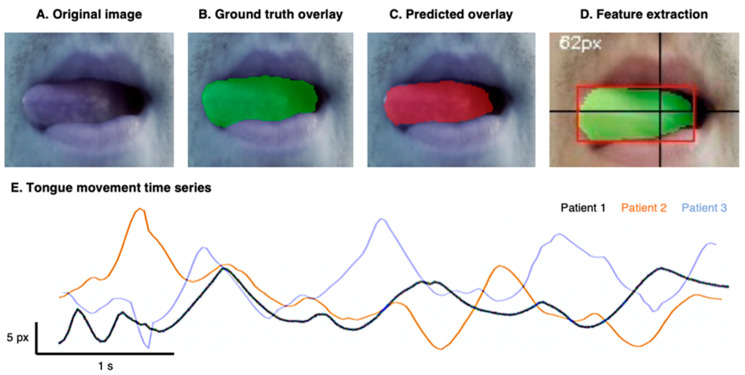
Automated tongue segmentation and feature extraction. (**A**) Original video frame. (**B**) Ground truth segmentation overlay. (**C**) Predicted segmentation obtained from the trained U-Net++ model. (**D**) Feature extraction showing the detected tongue region (red bounding box) and spatial calibration used to compute tongue displacement amplitude. (**E**) Time series of tongue movements during the task for three different patients.

The analysis workflow is illustrated in Figure 2. Each video frame was processed using a deep-learning segmentation model to isolate the tongue region (see next section for details). The tongue-tip position, operationally defined as the most lateral point of the segmented binary mask along the horizontal axis, was automatically tracked across frames, generating a two-dimensional time series of lateral displacement during the task.

Quantitative features were extracted from this displacement signal, derived directly from the raw smartphone video recordings. Two kinematic feature indexes were derived: (i) movement frequency, defined as:

$$Frequency\;\left(Hz\right)=\frac{Ncycles}{T}$$

where *Ncycles* represents the number of complete lateral oscillatory cycles detected within the recording and *T* = 5 s corresponds to the task duration; and (ii) maximum amplitude, defined as the greatest lateral displacement achieved during the task (in millimeters). These features were selected because they directly reflect tongue motor speed and range of motion and are readily interpretable in a clinical context. To account for interindividual variability in anatomy and movement range, frequency values were normalized by the maximum movement amplitude, such that all analyses were performed on the resulting normalized frequency. Normalized frequency was computed as:

$$Normalized\;Frequency\;\left(Hz/mm\right)=\frac{Frequency\;(Hz)}{Maximum\;Amplitude\;(mm)}$$

Inspection of amplitude distributions confirmed the absence of near-zero values that could artificially inflate the normalized ratio (see Appendix A.4). Each participant performed the task twice, and the mean value of both trials was used for analysis.

### 2.4. Tongue Segmentation Model

A standard UNet++ convolutional neural network [23] was used to segment the tongue region in video frames. The model was trained on a heterogeneous dataset of 350 manually annotated tongue images collected from online sources and participants (both patients and controls; see the rationale in the Appendix A), ensuring broad variability in tongue characteristics, illumination, and acquisition conditions. All images and corresponding segmentation masks were resized to a fixed spatial resolution of 256 × 256 pixels, pixel intensities were normalized to the [0, 1] range, and the data were processed into aligned arrays of inputs and binary ground-truth labels.

To rigorously evaluate segmentation performance and prevent data leakage, model training and evaluation were conducted using a 5-fold cross-validation strategy. The dataset was randomly partitioned into five mutually exclusive folds; in each iteration, four folds were used for training and one-fold for validation, such that every image served as validation data exactly once. Filenames were tracked to ensure that no images from the same participant were included in both training and validation subsets within a given fold. Importantly, the segmentation model was trained solely to optimize pixel-level mask accuracy and did not use diagnostic labels (ALS versus control) during training. Kinematic feature extraction and group-level statistical analyses were conducted only after segmentation training and validation were completed, thereby preventing label-driven information leakage into downstream analyses.

Model training was performed using TensorFlow/Keras for 100 epochs with the Adam optimizer (learning rate 1 × 10^−4^) and a loss function combining binary cross-entropy with soft Dice loss, to promote pixel-wise accuracy and spatial overlap. A batch size of 16 was used. Model performance on each fold’s held-out validation set was quantified using the Dice coefficient and Intersection over Union (IoU), two widely used measures that capture the degree of overlap between predicted and true masks [24]. Final performance values were computed by averaging metrics across all folds.

Representative examples of the original smartphone video frames, together with their corresponding segmentation outputs and extracted motion trajectories, are shown in Figure 2 to illustrate the transformation from raw input signals to quantitative features. The UNet++ was selected due to its demonstrated performance in medical image segmentation and its ability to generalize under heterogeneous acquisition conditions [23]. Because segmentation served as an intermediate step rather than a primary outcome, the methodological emphasis was placed on stability, reproducibility, and clinical applicability rather than on a comparison between distinct segmentation architectures.

Following segmentation, tongue motion trajectories were derived from the sequence of segmented masks, from which movement frequency (number of complete lateral cycles) and maximum amplitude (greatest lateral displacement) were extracted over 5-s intervals (see Section 2.3 for details). A detailed description of dataset construction, preprocessing, model architecture, and training procedures is provided in the Appendix A.

### 2.5. Statistical Analysis

Data analysis was performed using Python version 3.11.2 (Python Software Foundation). For the significance level, α = 0.05 was considered. Descriptive statistics are presented as frequencies and proportions for categorical variables, and as mean ± standard deviation (SD) for continuous variables.

To compare mean values, parametric tests such as the two-sample *t*-test or the one-way ANOVA were applied. If the normality assumption of a continuous variable was violated (significant Kolmogorov–Smirnov test with an absolute skewness > 2), non-parametric tests such as Mann–Whitney U-test or Kruskal–Wallis test were considered and the respective results reported, if non-consistent or different from parametric analysis. These analyses were used to assess differences in task performance between ALS patients and healthy controls, and within the ALS cohort between patients with and without bulbar symptoms, dysarthria, or dysphagia.

To explore clinical predictors of speech impairment, a multiple linear regression model was fitted with the ALSFRS-R speech subscore as the dependent variable, and age, tongue movement normalized frequency, and the presence of fasciculations, tongue “spasticity” on clinical assessment, and jaw-jerk reflex as independent variables. Model assumptions of normality, homoscedasticity, and multicollinearity were verified prior to interpretation. Because the ALSFRS-R speech item is ordinal (0–4), a sensitivity analysis was conducted using ordinal logistic regression in addition to linear regression. However, linear regression results are presented for ease of interpretability and comparability with prior literature. Statistical analyses were hypothesis-driven and based on clinically motivated comparisons. Given the limited number of primary comparisons and absence of exploratory variable selection, formal correction for multiple testing was not applied. Finally, to assess model stability and mitigate risk of overfitting, bootstrap resampling with 1000 iterations was performed to estimate optimism-corrected performance metrics. Ninety-five percent confidence intervals were calculated for all regression coefficients to provide estimates of precision.

## 3. Results

### 3.1. Participants

The demographic and clinical characteristics of participants are shown in Table 1. Groups had no significant differences in terms of age or sex distribution. There were no statistically significant differences between patients with and without bulbar dysfunction, in terms of age, sex, or disease duration (all *t*-tests with *p* > 0.05).

### 3.2. Model Performance

The U-Net++ model achieved high performance in tongue segmentation, with a Dice coefficient of 0.82 ± 0.04 and an IoU of 0.76 ± 0.06, indicating strong correspondence between predicted and ground-truth masks. These results demonstrate effective generalization and confirm the suitability of the architecture for precise medical image segmentation, ensuring that the extracted variables are reliable for the following analyses. For each participant, segmented masks and tongue-tip trajectories were visually inspected across frames, and the automatically detected oscillatory cycles were confirmed against the original video. This procedure ensured that the computed frequency values accurately reflected the true number of lateral tongue movements performed during the task. No cases of incorrect cycle detection were identified.

### 3.3. Task Performance

**Figure 3:**
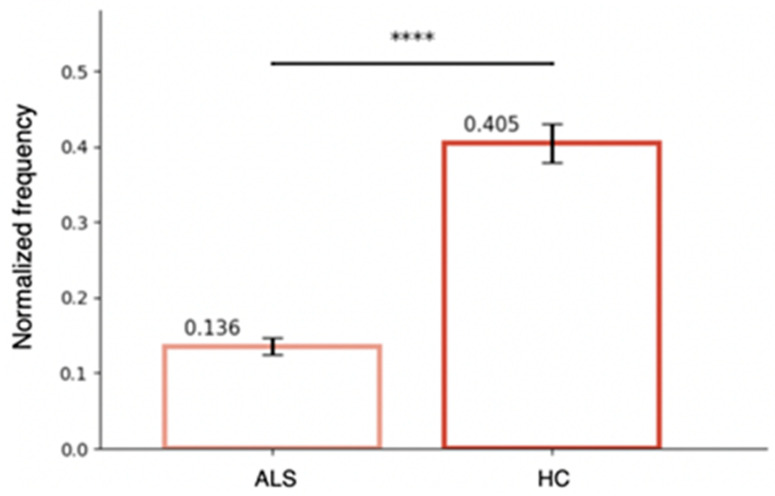
**Normalized tongue movement frequency in ALS patients and healthy controls (HC).** Bar plot showing the mean normalized frequency of tongue lateralization movements during a 5-s task. Patients with ALS exhibited significantly lower movement frequencies compared to healthy controls (ALS: mean 0.138; HC: mean 0.395; **** *p* < 0.0001).

A significant reduction in the normalized frequency of tongue movements was observed in ALS patients compared to healthy controls. Nonparametric analyses yielded the same significance pattern; therefore, parametric results are reported. As shown in Figure 3, the mean frequency of movements in the ALS group was markedly lower than in the control group (0.14 vs. 0.40, *t*= −9.58, *p* < 0.001). This difference indicates that ALS patients exhibit a substantial impairment in their ability to sustain rapid alternating tongue movements.

### 3.4. Relationship with Bulbar Impairments

**Figure 4:**
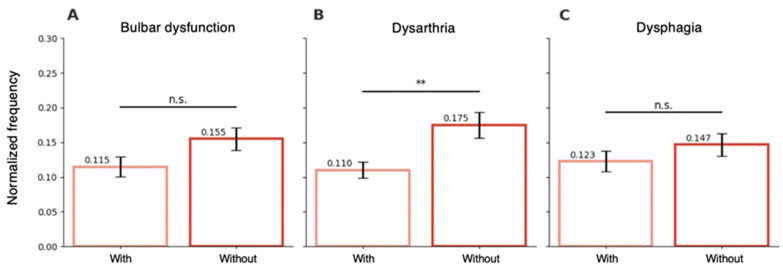
**Associations between bulbar symptoms and normalized frequency of tongue movements.** (**A**) Comparison between patients with and without bulbar dysfunction. No significant difference was observed. (**B**) Comparison between patients with and without dysarthria. Patients with dysarthria exhibited significantly lower normalized frequency (*t* = −3.13, ** *p* = 0.003). (**C**) Comparison between patients with and without dysphagia. No significant difference was observed.

In the ALS cohort, the presence of bulbar dysfunction (defined by the ALSFRS-R bulbar subscore) was not associated with significant differences in normalized frequency (0.155 in patients without bulbar dysfunction vs. 0.115 in those with bulbar dysfunction, *p* = 0.076.; Figure 4A). When different types of bulbar symptoms were analyzed separately, patients with dysarthria showed a significantly lower normalized frequency compared to those without dysarthria (0.110 vs. 0.175, *p* = 0.003; Figure 4B). In contrast, the presence of dysphagia was not significantly associated with normalized frequency (0.123 vs. 0.147, *p* = 0.30; Figure 4C). The regression model assessing clinical predictors of speech impairment accounted for 50.8% of the variance in speech scores (*R* = 0.713, R^2^ = 0.508, adjusted *R*^2^ = 0.43, *N* = 37; Table 2). In this model, normalized frequency was a significant predictor (β = 4.68, SE = 1.69, *t* = 2.77, *p* = 0.009), indicating that lower values predicted dysarthria severity. Similarly, tongue “spasticity” was significantly associated with lower speech scores (β= –0.64, SE = 0.25, *t* = –2.57, *p* = 0.015), suggesting that its presence was also a predictor of dysarthria severity. Ninety-five percent confidence intervals for regression coefficients are reported in Table 2. Bootstrap internal validation demonstrated minimal optimism in model performance estimates, with adjusted R^2^ values remaining stable following correction. These findings suggest reasonable internal stability within the constraints of the sample size. Age, presence of tongue fasciculations, and a brisk jaw jerk reflex were not significant predictors (*p* > 0.20). Moreover, the direction and statistical significance of the association between normalized frequency and speech score remained consistent between the ordinal logistic regression and linear regression, supporting robustness of the findings. All variance inflation (FIV) values were lower than 2, indicating no multicollinearity concerns.

## 4. Discussion

This study developed and validated a simple, smartphone-based clinical task for the objective assessment of tongue movements in patients with ALS, using a deep-learning segmentation model to quantify motion parameters. The method demonstrated robust segmentation performance and strong clinical relevance, revealing significant alterations in tongue movement dynamics in patients with ALS compared with healthy controls. This is particularly relevant because there are no accessible objective tools for assessing tongue movements, and evaluations still depend on the subjective judgment of neurologists, whether less or more experienced. Table 3 provides a brief overview of techniques currently used to assess bulbar motor control.

This work validated the feasibility of the task and evaluated its sensitivity to assess bulbar impairment.

From a physiological perspective, the tongue represents a particularly informative model for studying bulbar motor control and its vulnerability to neurodegeneration. It is composed primarily of skeletal muscle fibers without compartmentalization and rigid support, and thus it requires precisely coordinated activation of multiple muscle groups to generate movement and maintain structural integrity [31,32]. Voluntary lingual movements are mediated by a distributed, notably bilateral cortical network that exerts top-down modulation (namely in task-specific adaptation of motor sequences) over the motor control centers present in the brainstem [33]. From here, the hypoglossal nerve innervates the muscles of the tongue [34]. Because its movements depend heavily on fine hypoglossal motor-unit control, changes in movement speed, smoothness, coordination and range can serve as sensitive markers of bulbar dysfunction [35]. However, the tongue’s movement and shape change are determined not only by the internal and external forces generated by its intrinsic and extrinsic muscles [31], but they are also influenced by the reaction forces from surrounding structures, such as the bones, teeth, and food bolus [36]. This implies caution when interpreting the neuronal correlates of tongue movements. Additionally, the tongue exhibits translation along all three cardinal axes—protraction-retraction, elevation-depression, and lateral displacement—accompanied by three-dimensional shape deformations. For this specific task, we anticipated changes primarily in lateral movement rather than in shape or movement angles. This aspect made it sensitive to certain bulbar symptoms. For example, it is well studied that protraction and retraction of the tongue are both fundamental for swallowing by transporting food through the oral cavity [36,37] due to the activity of the genioglossus muscle. Understanding this physiological basis has direct implications for quantifying motor impairment in ALS.

In the present study, reduced normalized frequency of lateral tongue movements in the ALS group predicted dysarthria, but not dysphagia. This dissociation suggests that lateral movement frequency preferentially indexes speech-related motor impairment, rather than swallowing dysfunction. This specificity likely reflects fundamental differences in the motor demands of these behaviors: speech production requires precise, spatially distributed tongue positioning and localized morphological changes—kinematic features closely aligned with our experimental task—whereas swallowing depends primarily on anteroposterior translation with relatively stereotyped movement patterns [38,39].

At the same time, tongue “spasticity” as defined on clinical examination emerged as a significant predictor of speech performance, consistent with the clinical understanding that UMN involvement contributes to reduced tongue agility and strained–spastic speech patterns. The combined effects of lower normalized frequency and “spasticity” therefore capture similar aspects of bulbar motor dysfunction, particularly slowing and rigidity, both of which underlie deteriorating speech clarity in ALS. These findings further validate the clinical utility of subjective “spasticity” assessment, demonstrating that when combined with objective kinematic measures of lateral movement frequency, both metrics provide robust prediction of speech severity impairment.

From a technical perspective, the approach was well tolerated by patients, causing neither burden nor noticeable fatigue. Its simplicity highlights the suitability for scalable deployment as it requires no specialized equipment, minimal training, and can be recorded using any contemporary smartphone. This lowers the barrier to adoption in routine clinical practice, even in non-specialized settings. Because segmentation and feature extraction are fully automated, clinicians can receive objective metrics without performing manual annotations or calculations, while also reducing observer biases. As such, the method can be rapidly integrated into research cohorts, clinical trials, and standard follow-up visits.

These properties also establish a foundation for remote and longitudinal monitoring. Given the heterogenous and often rapid progression of ALS, tools capable of detecting subtle within-patient changes are needed [17]. Our results show that lateral tongue movement normalized frequency captures interindividual variation in bulbar motor control; the key next step is determining whether this metric can detect within-subject decline before conventional clinical scales change. Longitudinal home-based recordings will allow us to characterize individual trajectories, identify early inflection points, and develop predictive models of bulbar deterioration. Such capabilities could substantially improve proactive clinical management, refine prognostic accuracy, and enhance the sensitivity of outcome measures used in therapeutic trials targeting bulbar function.

This work had some limitations, including the relatively small sample size, the video resolution, and the potential influence of chin movements accompanying tongue motion. The relatively small ALS cohort limits the complexity of multivariable modeling. Although internal validation using bootstrap resampling suggested reasonable model stability, external validation in an independent cohort will be essential before considering clinical deployment. The resolution of the smartphone video recordings directly affects the accuracy of amplitude measurements, as insufficient detail may obscure the exact frame in which peak displacement occurs. Nevertheless, modern smartphones typically offer high-quality video capture, which minimizes this issue. Furthermore, the algorithm consistently identifies the frequency of lateral tongue movements, suggesting that the overall reliability of the measurements remains robust despite these constraints. Although formal manual re-annotation was not performed, comprehensive visual verification of segmentation outputs and derived kinematic signals across all participants supported the reliability of frequency estimation. Future work may incorporate quantitative error propagation analyses to further characterize robustness. Regarding chin movements, we observed that some patients move the chin simultaneously and in parallel with the tongue. A potential direction for future work is to systematically record these chin displacements and incorporate them into the model, allowing us to evaluate their contribution to, and influence on, speech performance. Moreover, future iterations of the pipeline may incorporate automated facial landmark tracking and frame-to-frame alignment to further enhance robustness.

Another promising idea is to analyze tongue protraction movements, assess their maximum amplitude, and investigate how these metrics relate to bulbar symptomatology. Finally, although this was not the primary goal, the tongue-segmentation model may have limited generalization to other populations, as it was trained on a relatively small dataset and fine-tuned specifically for our cohort. We chose this approach to ensure that the extracted features were accurate, allowing for fully reliable clinical analyses. Comparisons across alternative segmentation architectures and tracking strategies, especially when trained on larger and more diverse datasets, could further improve generalization performance. Such improvements may be particularly beneficial for the robust assessment of more sensitive tongue movement metrics.

## 5. Conclusions

In this study, we developed and validated a simple smartphone-based task combined with deep-learning segmentation to objectively quantify tongue movements in ALS, demonstrating robust technical performance and strong clinical relevance. ALS patients showed markedly reduced lateral tongue movement frequency, which, together with clinically identified tongue “spasticity”, independently predicted speech impairment, highlighting the specificity of this measure for detecting speech motor dysfunction. The task was well tolerated, required minimal equipment, and could be easily implemented at home, supporting its potential for scalable, personalized, and remote monitoring of bulbar function. Overall, this work provides a minimally invasive and accessible tool that complements existing assessments and may enhance early detection and tracking of bulbar deterioration in ALS.

## Figures and Tables

**Table 1:** Clinical characteristics of ALS patients and controls.

Characteristic	ALS Patients (*N* = 37)	Controls (*N* = 20)
**Age (mean ± SD)**	63.9 ± 12.4	61.1 ± 9.1
**Gender**		
Men	17 (46%)	10 (50%)
Women	20 (54%)	10 (50%)
**Symptom duration (months)**		
Median	23.5	-
1st–3rd IQR	9.80–38.96	-
**Disease onset**		
Bulbar onset	11 (30%)	-
Upper limb onset	16 (40%)	-
Lower limb onset	11 (30%)	-
**ALSFRS-R total score (0–48) (mean ± SD)**	36 ± 6.7	-
**Bulbar dysfunction**	17 (46%)	-
**Dysphagia**	3.4 ± 0.9	-
**Dysarthria**	3.2 ± 0.8	-
**Tongue spasticity**	11 (30%)	-
**Tongue fasciculations**	14 (38%)	-
**Abnormal jaw jerk**	7 (19%)	-

ALSFRS-R = revised amyotrophic lateral sclerosis functional rating scale; IQR = interquartile range.

**Table 2:** Model fit and regression coefficients for dysarthria.

Model Fit Statistics					
Model	*R*	*R* ^2^	Adjusted *R*^2^		
1	0.713	0.508	0.430		
**Regression coefficients—Dysarthria score**					
*Predictor*	*Estimate*	*SE*	*t*	*p*	*95% CI*
**Intercept**	**3.34727**	**0.67678**	**4.946**	**<0.001**	**1.96–4.73**
Age	−0.0074	0.00889	−0.827	0.415	−0.026–0.011
**Normalized frequency**	**4.68230**	**1.69174**	**2.768**	**0.009**	**1.23–8.13**
**Tongue spasticity (yes-no)**	**−** **0.642124**	**0.24977**	**−** **2.571**	**0.015**	**−** **1.15–** **−** **0.13**
Tongue fasciculations (yes-no)	−0.21589	0.25142	−0.859	0.397	−0.73–0.30
Abnormal jaw jerk (yes-no)	−0.29142	0.29251	−0.996	0.327	−0.89–0.31

Note. Model estimated using sample size of *N* = 37. Bold represents a statistically significant *p*-value.

**Table 3:** Overview and comparison of techniques used to assess bulbar motor control. The table summarizes the primary targeted bulbar function, patient burden, required resources, suitability for remote monitoring, and key limitations of commonly used approaches [12,25,26,27,28,29,30].

Method	Targeted Bulbar Function	Patient Burden	Required Resources	Suitability for Remote Monitoring	Key Limitations
**Electromyography (EMG)**	Lower motor neuron involvement; muscle denervation	High (invasive, uncomfortable)	EMG system; trained neurophysiologist	No	Invasive, limited repeatability, not feasible for frequent follow-up
**Speech/cough analysis**	Dysarthria, speech intelligibility, and swallowing	Low	Microphone; signal processing expertise or automated analysis pipeline	Yes	Indirect assessment of tongue motor control; influenced by language, cognition, and compensatory strategies
**Lingual pressure measurements**	Tongue strength during swallowing	Moderate	Dedicated pressure sensors; trained personnel	Limited	Focuses on strength rather than movement dynamics; limited availability
**Imaging-based methods (e.g., MRI, ultrasound)**	Tongue structure and kinematics	Moderate to high	Specialized imaging equipment; expert analysis	No	Costly, time-consuming, not scalable for routine or remote use
**Proposed smartphone-based tongue movement assessment**	Voluntary tongue motor control related to speech	Low	Smartphone or video camera; automated analysis pipeline	Yes	Dependent on the segmentation model performance

Bold represents different methods.

## Data Availability

The study data will be made available upon reasonable request to the corresponding author.

## Abbreviations

The following abbreviations are used in this manuscript:

**ALS**	Amyotrophic lateral sclerosis
**ALSFRS-r**	Revised Amyotrophic Lateral Sclerosis Functional Rating Scale
**UMN**	Upper motor neuron
**LMN**	Lower motor neuron
**IoU**	Intersection over Union
**ANOVA**	Analysis of variance
**SD**	Standard deviation
**IQR**	Interquartile range
**Hz**	Hertz
**RGB**	Red, green, blue
**BCE**	Binary cross-entropy

## Appendix A

### Appendix A.1. Dataset Construction

A heterogeneous dataset of tongue images was compiled from two sources: publicly available images obtained through the Kaggle website [40], and photographs collected directly from study participants. In total, 350 images of varying resolutions, colors, and acquisition conditions were manually annotated using the LabelMe tool [41]. Heterogeneity in tongue characteristics, illumination, and viewpoint was intentionally preserved to improve the robustness and generalization of the segmentation model. To ensure that the model would perform reliably on the images acquired for our experimental task, 50 images of the dataset were extracted from video frames recorded during the study (balanced across control participants and patients). Including these task-specific samples allowed the model to adapt to the visual characteristics of our acquisition setup, thereby increasing the fidelity of downstream metric extraction. Importantly, these task-derived images were single frames extracted from distinct videos, rather than consecutive frames from the same video segment, thereby minimizing redundancy and reducing the risk of near-duplicate samples.

All images and their corresponding segmentation masks were resized to 256 × 256 pixels and represented as three-channel (RGB) arrays. Masks were converted to binary format, expanded to a single-channel tensor to match model requirements, and normalized to the range [0, 1] alongside the input images. The resulting dataset was stored as three aligned arrays—images, masks, and filenames—allowing subsequent verification that no image was inadvertently shared across training and validation sets. To rigorously assess model performance and prevent data leakage, we employed a 5-fold cross-validation procedure. Using KFold (five splits, shuffled, random state = 40), each fold produced disjoint training and validation subsets. When multiple images originated from the same participant, they were assigned to the same fold (participant-level grouping) to ensure that no participant contributed images to both training and validation subsets within a given split. For every split, filename comparison ensured that no sample was present in both subsets. This approach enabled robust assessment of segmentation accuracy while maximizing the use of available data. Moreover, the majority of the dataset consisted of independent online images, while the task-derived subset represented a minority portion.

### Appendix A.2. Tongue Segmentation Model Architecture

A U-Net++ convolutional neural network was implemented for dense tongue segmentation. The architecture follows the nested and densely skip-connected design introduced by Zhou et al. [23], which enhances gradient propagation and enables multiscale feature refinement throughout the decoder pathway. Input images of size 256 × 256 × 3 pass through an encoder composed of five levels. Each level contains a convolutional block comprising two sequential 3 × 3 convolutional layers with ReLU activation and “same” padding, followed by a 2 × 2 max-pooling operation (except at the deepest layer). The numbers of filters at successive encoder depths were 32, 64, 128, 256, and 512. The decoder mirrors the encoder but incorporates the characteristic nested skip pathways of U-Net++: each decoder node receives (i) the up sampled output of the deeper decoder stage and (ii) aggregated encoder- level features and intermediate decoder features from parallel semantic depths. These feature maps are concatenated channel-wise before entering the convolutional blocks. Upsampling between decoder levels is performed using bilinearUpSampling2D operations. A final 1 × 1 convolution layer with sigmoid activation maps the refined decoder features to a per-pixel probability of tongue presence, producing an output mask of size 256 × 256 × 1. This architecture supports fine-grained localization while retaining contextual information from deeper semantic layers.

### Appendix A.3. Model Compilation, Training, and Prediction

The segmentation model was implemented in TensorFlow/Keras [42] and trained using the Adam optimizer [43] with a learning rate of 1 × 10^−4^. To jointly optimize pixel-wise accuracy and spatial overlap, we employed a composite loss function that combines binary cross-entropy (BCE) with a soft Dice loss. Because the tongue typically occupies a small fraction of each image, the Dice component mitigates foreground–background class imbalance and stabilizes training. Model performance was monitored using the Dice coefficient and Intersection over Union (IoU), computed by thresholding prediction probabilities at 0.5.

Training was performed for 100 epochs per cross-validation fold with a batch size of 16. To improve robustness to anatomical variability, pose, and imaging conditions, we applied extensive online data augmentation using the *tf.data* pipeline. Augmentations included random horizontal and vertical flips, rotations of 0°, 90°, 180°, or 270°, zoom perturbations in the range 0.9–1.1×, brightness and contrast modulation, hue and saturation jitter, and additive Gaussian noise (*σ* = 0.02). All geometric transformations were applied identically to images and masks, and masks were re-binarized when necessary to preserve label integrity.

For each fold, a new model instance was trained from scratch and evaluated on the corresponding held-out validation set. Predictions were generated via forward inference and binarized to obtain segmentation maps. Per-image Dice and IoU metrics were computed to quantify performance. Final results are reported as the mean ± standard deviation across folds, providing an unbiased estimate of the model’s generalization performance.

### Appendix A.4. Automated Variable Extraction Algorithm

An automated algorithm was developed to quantify tongue motion from the segmented video frames. For each frame, the binary mask generated by the U-Net++ model was processed to extract the tongue contour, and the tongue tip was identified as the most anterior point of the contour (maximum horizontal coordinate in right-facing recordings, or minimum in left-facing ones). The two-dimensional coordinates of the tongue tip were tracked across all frames. The maximum amplitude of movement was calculated as the largest Euclidean distance between any two detected positions, corresponding to the peak-to-peak displacement of the tongue tip. The frequency of lateral movement was determined by analyzing the horizontal displacement trajectory of the tongue tip over the 5-s video and identifying the number of complete oscillatory cycles, defined as successive left–right–left (or right–left–right) movements. The total number of cycles was divided by the video duration to obtain the movement frequency in hertz (Hz). We then converted pixel distances to millimeters using a known spatial reference from the imaging setup.

To convert pixel distances into millimeters (mm), a physical spatial reference marker measuring exactly 10 mm was positioned immediately below the participant’s lower lip and was visible in all recordings. This marker was placed in approximately the same frontal plane as the tongue to minimize perspective distortion. For each recording, the marker length was measured in pixels from a stable reference frame, and a participant-specific scaling factor (mm per pixel) was computed using the known 10 mm length. This scaling factor was then applied to all displacement measurements within that recording. This per-recording calibration procedure compensates for moderate variations in camera-to-face distance (approximately 15 cm) and minor framing differences, as any uniform scaling caused by distance variation proportionally affects both the marker and tongue displacement. Recordings were obtained in near-frontal orientation by a trained neurologist to further minimize geometric distortion. Because the task primarily involved lateral movements occurring in the image plane, residual out-of-plane perspective effects were considered minimal. Amplitude values are therefore reported in millimeters (mm), and movement frequency in hertz (Hz). To further account for interindividual differences in movement range and reduce sensitivity to residual scaling variability, frequency values were normalized by maximum amplitude prior to statistical analyses.
